# First Covid-19 Vaccination Rates Among Pregnant Tennessee Women by Month of Conception and Urbanization Level: March 2021 – November 2021

**DOI:** 10.13023/jah.0801.07

**Published:** 2026-04-01

**Authors:** Claudia Valenzuela, Anne K. Driscoll, Elizabeth Harvey, Heather Wingate

**Affiliations:** National Center for Health Statistics, Centers for Disease Control and Prevention; National Center for Health Statistics, Centers for Disease Control and Prevention; Tennessee Department of Health; Tennessee Department of Health

**Keywords:** Appalachia, COVID-19, pregnancy, urbanization level, vaccination

## Abstract

**Introduction:**

Throughout the pandemic, COVID-19 vaccination coverage was lower for the general population living in rural counties than in urban counties. In Tennessee (TN), COVID-19 vaccinations became available to pregnant women in March 2021. Little is known about rates of COVID-19 vaccination in pregnancy during the pandemic, or whether these rates changed or differed by urbanization level.

**Purpose:**

The purpose of this study is to describe COVID-19 vaccination rates among pregnant women in TN by month of conception and urbanization level.

**Methods:**

COVID-19 vaccination data from the TN Immunization Information System and the TN Surveillance for Emerging Threats to Mothers and Babies Network for women who conceived between March – November 2021 were linked to birth data from the National Center for Health Statistics. Women were categorized based on the urbanization level of their county of residence.

**Results:**

Vaccination rates during pregnancy were highest for women in large central metropolitan counties and lowest for women in rural counties. Among women who conceived in March 2021, those living in urban counties were 1.5 – 2 times as likely to be vaccinated as those living in rural counties. From March – November 2021, declines in vaccination rates were observed for all urbanization levels with larger declines in urban counties. There was no significant difference in vaccination rates across all urbanization levels among women who conceived in November 2021.

**Implications:**

Differences noted in vaccination rates, particularly at the beginning of an outbreak, can help inform decision-makers on where and how to direct vaccination resources.

## INTRODUCTION

Previous reports have shown the adverse impact of COVID-19 on maternal and newborn health, including higher risk for outcomes such as admission to an intensive care unit, neonatal intensive care unit, and preterm birth.^1^–^5^ Throughout the pandemic, COVID-19 vaccination coverage was lower for the general population living in rural counties than in urban counties.^6^–^7^ In Tennessee (TN), COVID-19 vaccinations became available to pregnant women in March 2021.^8^ Little is known about rates of COVID-19 vaccination in pregnancy during the pandemic,^9^–^10^ or whether these rates changed or differed by urbanization level. This study describes rates of COVID-19 vaccination during pregnancy among TN women with a live birth who conceived from March 2021 – November 2021 by month of conception and urbanization level.

## METHODS

COVID-19 vaccination data from the Tennessee Immunization Information System (TennIIS) (COVID-19 vaccine status and dates of vaccination) linked to data from the TN Surveillance for Emerging Threats to Mothers and Babies Network (birth certificate numbers, month of birth, and year of birth) for women who became pregnant between March 2021 – November 2021 were provided to the National Center for Health Statistics (NCHS). Births from the TN dataset were linked to births from the NCHS vital statistics birth file to allow for the analysis of additional variables such as county of residence, maternal race and ethnicity, and maternal age.^11^ Counties were classified according to their urbanization status using the NCHS Urban-Rural Classification Scheme and merging the county of residence geographic federal information processing standard (FIPS) codes with county-level FIPS codes from the 2013 NCHS Urban-Rural Classification Scheme data set.^12^ Women were categorized based on the urbanization level of their county of residence in TN: large central metropolitan (LCM), large fringe metropolitan (LFM), medium or small metropolitan (MSM), and micropolitan or non-core. The three metropolitan urbanization levels (LCM, LFM, and MSM) are classified as urban counties, and the micropolitan or non-core counties are classified as rural.

COVID-19 vaccination was defined as the receipt of a first dose of a COVID-19 vaccine during pregnancy. Women who received at least one dose of vaccine prior to conception were excluded from the analysis. Date of birth of the infant and the obstetric estimate of gestation were used to estimate the date of conception and the timing of vaccination.

Vaccination rates during pregnancy for all TN births are presented for women who became pregnant between March 2021 – November 2021 and had a live birth by the end of August 2022 by month of conception and urbanization level.

The differences between rates noted in the text are based on an independent two sample t-test and are statistically significant at the 0.05 level unless otherwise noted. References to trends in rates were evaluated using the JoinPoint Regression Program.^13^

## RESULTS

In TN, 60,199 women conceived between March 2021 – November 2021 and had a live birth by the end of August 2022. Among these women, 14,864 (24.7%) received their first COVID-19 vaccination before their pregnancy, leaving 45,335 women eligible to receive a first vaccination during pregnancy. Of these women, 15.9% received a first vaccination during pregnancy (Table 1). Vaccination rates during pregnancy were highest for women in the LCM counties and lowest for women in rural counties; rates ranged from 19.5% of women in LCM counties, to 15.6% in LFM counties, 15.2% in MSM counties to 8.8% in rural counties (Table 1). The average age of women receiving their first COVID-19 vaccination during pregnancy was 29.1 years. The mean age was lowest for women in rural counties (27.4 years) and highest for those in LCM (29.6 years) and LFM (29.3 years) counties. Across all age groups, women in LCM counties were more likely to receive their first vaccination during pregnancy than those in rural counties. With the exception of women age 40 and older, those in MSM counties were more likely to be vaccinated than those in rural counties. Across all race and ethnicity groups, women in LCM counties were more likely to receive their first vaccination during pregnancy than those in rural counties. Among white, black and Hispanic women, those living in rural counties were less likely than those in all other urbanization levels to receive their first vaccination during pregnancy.

**Figure 1:**
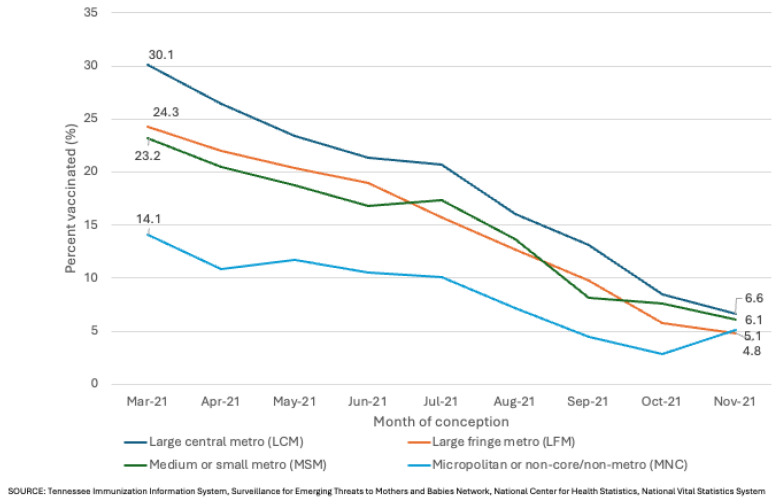
Covid-19 vaccination rates during pregnancy by month of conception and urbanization level among TN women who conceived between March 2021 – November 2021 NOTES: *SOURCE: Tennessee Immunization Information System, Surveillance for Emerging Threats to Mothers and Babies Network, National Center for Health Statistics, National Vital Statistics System

Among women who conceived in March 2021, women living in LCM counties had higher rates of vaccination during pregnancy than women in all other counties, while women in rural counties had lower rates than women in all other counties (Fig. 1). Additionally, women living in LCM counties were twice as likely to receive a vaccination during pregnancy (30.1%) as those living in rural counties (14.1%). In March 2021, vaccination rates across all urbanization levels were significantly different from one another except for rates among women living in LFM counties (24.3%) compared with women living in MSM (23.2%) counties. From March 2021 – November 2021, declines in COVID-19 vaccination rates were observed for all urbanization levels with larger declines in urban counties; those declines were greater for women who conceived in the latter part of the reporting period, July or August – November. For example, vaccination rates declined by 8%–10% on average per month from March – July or August for women living in LCM and LFM counties, and then by 23%-27% on average per month from July or August – November for women living in LCM, LFM, and MSM counties. Vaccination rates for women living in rural counties decreased by 14% on average per month from March – November. As a result of the larger declines among urban counties, there was no significant difference in vaccination rates across all urbanization levels among women who conceived in November 2021; rates among these women ranged from 4.8% to 6.6%.

## DISCUSSION

The analysis shows a decline in COVID-19 vaccination during pregnancy among both urban and rural counties in TN for women who conceived from March – November 2021. Although our data show that vaccination rates were higher for pregnant women in urban counties who conceived in earlier months, we also found that vaccination rates declined more steeply among these women over time and that the urban-rural COVID-19 vaccination gap among pregnant women narrowed over time; by the end of the study period, November 2021, vaccination rates among pregnant women did not differ significantly by urbanization level. Our study period begins when COVID-19 vaccinations first became available, in early 2021. Declines in first vaccination during pregnancy noted over the study time period could be attributed, in part, to women receiving their first vaccination before pregnancy, thereby excluding them from our study; however, reasons for this decline could not be explored in these data. These results provide data on vaccine uptake among pregnant women in TN and potential disparities by urbanization at least in the earlier stages of vaccine availability. This study uniquely matches birth data with timing of COVID-19 vaccination and is not subject to reporting bias or response rates. This study has several limitations. The data are from one state and are not generalizable to other states or the entire U.S. The data do not include information on women’s beliefs or attitudes towards COVID-19 vaccines, nor does it include information on vaccine availability. Nevertheless, the results provide data on vaccine uptake among pregnant women in TN and potential disparities by urbanization at least in the earlier stages of vaccine availability.

## IMPLICATIONS

Data regarding availability of the vaccine per region and accessibility to health care services was not available for this study. Additionally, research that examines the beliefs and attitudes of pregnant women towards COVID-19 vaccines by urbanization level is needed to put these results into context. Such information, along with the data presented, can help medical professionals and decision-makers estimate vaccination rates among pregnant populations in urban and rural areas during emerging outbreaks. Differences noted in vaccination rates, particularly at the beginning of an outbreak, can help inform decision-makers on where and how to direct vaccination resources.

**SUMMARY BOX**

**What is already known about this topic?**

Throughout the pandemic, COVID-19 vaccination coverage was lower for the general population living in rural counties than in urban counties. Previous reports have shown the adverse impact of COVID-19 on maternal and newborn health.

**What is added by this report?**

These results provide data on vaccine uptake among pregnant women in TN and potential disparities by urbanization at least in the earlier stages of vaccine availability. This study uniquely matches birth data with timing of COVID-19 vaccination and is not subject to reporting bias or response rates.

**What are the implications for future research?**

Data identifying vaccine availability per region, accessibility to health care services, and beliefs and attitudes of pregnant women towards COVID-19 vaccines by urbanization level are needed to help estimate vaccination rates among pregnant populations in urban and rural areas during emerging outbreaks.

## Figures and Tables

**Table 1:** Characteristics of TN mothers who received a first COVID-19 vaccine during pregnancy by urbanization level

	Total		Large Central Metro (LCM)		Large Fringe Metro (LFM)		Medium/Small Metro (MSM)		Micro/Non-Core (MNC)	
N	45,335		15,497		7,121		16,769		5,948	
	Percent	CI	Percent	CI	Percent	CI	Percent	CI	Percent	CI
Vaccinated during pregnancy	15.9	15.6–16.2	19.5	19.1–19.9	15.6^b^	14.8–16.4	15.2	14.7–15.7	8.8	8.1–9.5
**Maternal Characteristics**										
**Age**										
Younger than 20	8.9	7.9–9.9	9.8^a^^b^	8.8–11.6	9.0^b^	6.3–11.7	9.5	7.8–11.2	5.8	3.9–7.7
20–24	11.2	10.6–11.8	14.1	13.0–15.2	9.6	8.2–11.0	11.5	10.6–12.4	6.8	5.7–7.9
25–29	15.9	15.3–16.5	18.5^a^	17.4–19.6	17.0	15.4–18.6	15.0	14.0–16.0	10.6	9.2–12.0
30 and older	20.6	20.0–21.2	24.9	23.8–26.0	19.0^b^	17.5–20.5	19.5	18.5–20.5	10.3	8.8–11.8
30–34	19.8	19.0–20.6	23.9	22.6–25.2	18.0^b^	16.2–19.8	19.1	17.9–20.3	10.5	8.7–12.3
35–39	21.7	20.5–22.9	26.2	24.2–28.2	20.5^b^	17.7–23.3	20.7	18.7–22.7	9.2	6.5–11.9
40 and older	23.1	20.5–25.7	29.2^a^	25.0–33.4	23.5^b^	17.0–30.0	17.8^c^	13.6–22.0	13.2	7.0–19.4
Mean age	29.1	28.9–29.2	29.6^a^	29.4–29.8	29.3	29.0–29.6	28.6	28.4–28.8	27.4	27.0–27.9
**Race and Ethnicity** **†**										
White, non-Hispanic	15.4	15.0–15.8	22.5	21.4–23.6	15.4^b^	14.4–16.4	14.8	14.2–15.4	8.6	7.8–9.4
Black, non-Hispanic	15.2	14.5–15.9	16.1^a^	15.2–17.0	15.1^b^	12.6–17.6	13.5	12.0–15.0	8.3	5.1–11.5
Asian, non-Hispanic	29.1	25.7–32.5	36.0^a^	30.6–41.4	27.0^b^^c^	19.8–34.2	22.9^c^	17.0–28.8	13.5	2.5–24.5
Hispanic	17.8	16.9–18.7	19.0^b^	17.6–20.4	15.3	13.1–17.5	19.6	17.8–21.4	10.9	8.5–13.3

NOTES:

* Race and Hispanic origin are reported separately on birth certificates; persons of Hispanic origin may be of any race. In this table, non-Hispanic women are classified by race. Race categories are consistent with the 1997 Office of Management and Budget standards. Single-race is defined as only one race reported on the birth certificate. Only the major race and Hispanic origin groups are presented, therefore, numbers may not add to 100.

† SOURCE: Tennessee Immunization Information System, Surveillance for Emerging Threats to Mothers and Babies Network, National Center for Health Statistics, National Vital Statistics System

§ Legend:

a not significantly different from LFM (*p*<0.05)

b not significantly different from MSM (*p*<0.05)

c not significantly different from MNC (*p*<0.05)
